# Influences of Ag Addition on the Microstructure and Mechanical Properties of Al-4Mg Alloy

**DOI:** 10.3390/ma15217705

**Published:** 2022-11-02

**Authors:** Cheng Guo, Yifei Chen, Haitao Zhang

**Affiliations:** 1 College of Mechanical Engineering, Yanshan University, Qinhuangdao 066004, China; 2 Key Lab of Electromagnetic Processing of Materials Ministry of Education, Northeastern University, Shenyang 110004, China

**Keywords:** Al-4Mg alloy, Ag-additions, age hardening, nanoscale precipitates

## Abstract

In this study, the influence of Ag on the microstructures, precipitation behavior and mechanical properties of the Al-4Mg alloy are investigated. For the as-cast alloys, Ag can reduce the dimension of the precipitates and promote the precipitation of numerous fine-scale AlMgAg particles. In the aging process, Ag promotes the precipitation of nanoscale MgAg phase and T-Mg_32_(Ag, Al)_49_ phase, which improves the aging hardening response of the alloy. At the peak-aged stage of 210 °C, the ultimate tensile strength of the Ag-bearing alloy is 280 MPa, and the yield strength is 200 MPa, which is much higher than that of Ag-free alloy.

## 1. Introduction

Al-Mg alloys have the advantages of small density, good welding performance and excellent corrosion resistance [1]. In recent years, with the continuous improvement of the application demand, people have higher requirements on the performance of the Al-Mg alloy, especially its mechanical properties and corrosion resistance. Though the strength of Al-Mg alloys can be improved by aging strengthening, solution strengthening and work hardening, the β phase (Al_3_Mg_2_) was easy to precipitate along grain boundaries. Thus, the corrosion resistance of the alloy decreases when the content of Mg exceeds 3 wt.%. Therefore, the strength of the alloy was limited [2,3].

Aging strengthening is an important method to improve the strength of aluminum alloys. The Guinier Preston (GP) zones played a key role in the course of heat treatment strengthening. For Al-Mg alloys, due to the instability of the GP zones, it dissolved at room temperature and the nucleation sites of the precipitates were limited. In the artificial aging process of the Al-Mg alloys, only few coarse scale β’ or β phases can be detected [4,5,6]. Therefore, the aging strengthening effect was limited. Recently, some studies show that the addition of Sc in Al-Mg alloys can improve the properties of alloys evidently, as numerous nanoscale Al_3_Sc were drawn into the alloys [7,8]. However, Sc atoms were easy to precipitate in the process of hot working, and once precipitated it is hard to dissolve back into the matrix. Therefore, Sc was difficult to apply in solution-aging process. Some studies have reported that small amounts of Ag can induce age-hardening in Al-Mg alloys. The strengthening phase was thought to be a T phase (Mg_32_(Al, Ag)_49_) in Al-Mg-Ag alloys because the cell closely resembles those of the ternary phase Al_6_CuMg_4_ and (Mg_32_(Al, Zn)_49_ [9]. However, the evidence showed deficiency due to the restriction of detection technology at that time. In recent years, some research has reported that Ag played a positive role on aging hardening of an aluminum alloy [10,11]. For 5xxx aluminum alloys, the influence of Ag is mainly focused on the high-Mg content (>10 wt.%) Al-Mg alloys [12,13]. Due to the poor corrosion resistance and inferior processability, the application of high-Mg content Al-Mg alloys was limited. In addition, the content of Mg played a crucial role in the structure and component of precipitates. Therefore, it is essential to study the effects of Ag on the low-magnesium content Al-Mg alloys. In this paper, the influence of Ag on the initial as-cast microstructures, precipitation behavior and mechanical properties of low-magnesium content Al-Mg-Ag alloys is discussed in detail.

## 2. Experimental

Alloy ingots of the nominal composition Al-4wt.%Mg with or without 0.6 wt.% Ag (hereafter referred to as Al-4Mg-0.6Ag or Al-4Mg alloy) were prepared by medium frequency furnace using 99.8 wt.% Al, 99.99 wt.% Mg and 99.99 wt.% pure Ag. The size of each ingot was about 165 mm × 100 mm × 28 mm. After homogenization treatment at 500 °C for 24 h, alloy ingots were hot-rolled to 5 mm and cold-rolled to 2 mm. After that, the alloy plates were solution treated for 2 h at 500 °C followed by water-cooling, and then aged for 0–32 h at 120–240 °C, respectively.

The Leica DMI5000M-type optical microscope (OM) and SSX-550 scanning electron microscope (SEM) were used to analyze the microstructures of the two alloys. Energy dispersive spectrometer (EDS) was used to ensure the compositions of precipitate phases. All specimens for transmission electron microscopy (TEM) were thinned to perforate by a twin-jet electro polishing technique at −25 °C. The electrolyte was a solution of 33 vol.% HNO_3_ and 67 vol.% CH_3_OH. The aging microstructures were observed using Tecnai G^2^20 transmission electron microscope (TEM) operating at 200 kV. Tensile tests were carried out using CSS-44100 type universal testing machine with a crosshead speed of 2 mm/min. The width and length of gauge of specimens were 12.5 mm and 50 mm, respectively. The tensile test results of each sample were the average of three measurements. Micro-hardness was measured using Vickers hardness instrument at a load of 5 KG and loading time of 15 s. The hardness value of each sample had an average of 7 data points.

## 3. Results and Discussions

### 3.1. Microstructural Observations

**Figure 1:**
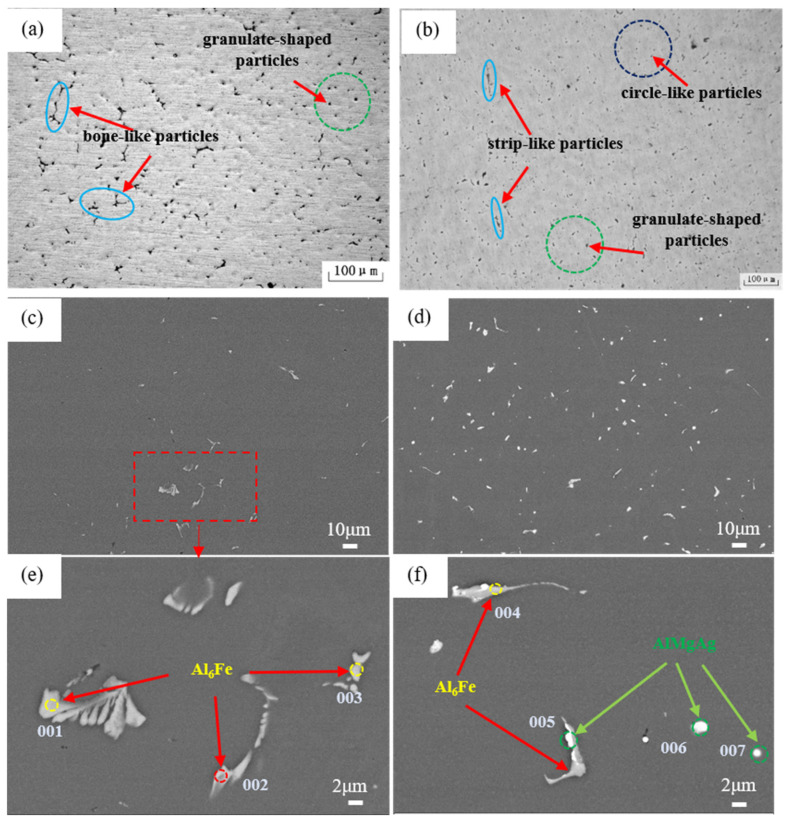
OM images of (**a**) as-cast Al-4Mg alloy, (**b**) as-cast Al-4Mg-0.6Ag alloy; (**c**,**e**) SEM pictures of Al-4Mg alloy, (**d**,**f**) SEM images of Al-4Mg-0.6Ag alloy.

Figure 1 represents the microstructures of the as-cast Al-4Mg alloys with or without Ag additions. For the Ag-free alloy, in Figure 1a,c, the microstructure contains many bone-like particles and granulate-shaped phases. The magnified morphology of the particles was shown in Figure 1e. Corresponding EDS results in Table 1 indicate that Al_6_Fe was the primary second phase in the as-cast Al-4Mg alloy [14]. However, after the addition of 0.6 wt.% Ag, the amount of the particles increases evidently, as shown in Figure 1b,d. According to Figure 1f and Table 1, the size of the Al_6_Fe decreases in a certain extent and some Ag adheres to the edge of Al_6_Fe phases during solidification. In addition, many fine-scale circle-like particles can also be detected in the as-cast microstructures of the Ag-bearing alloy. The EDS results in Table 1 illustrates these circle-like particles contain Al, Mg and Ag elements and can be defined as AlMgAg ternary phase.

For Al-4Mg alloy, as the solubility of Mg varies greatly with temperature, the faster cooling rate in the water-cooling copper mold can inhibit the precipitation of Al_3_Mg_2_. Most of Mg atoms dissolve into the Al-matrix. Therefore, Al_6_Fe particles are the primary second phases in the as-cast microstructures. However, for the Al-4Mg-0.6Ag alloy, Ag may act as the nucleation sites of Al_6_Fe particles and refine the size of these particles. For another, Ag and Mg atoms can reduce the solubility of each other and exhibit strong binding energy between them [15]. So numerous circle-like AlMgAg particles precipitate in the as-cast microstructures of the Al-4Mg-0.6Ag alloy.

**Figure 2:**
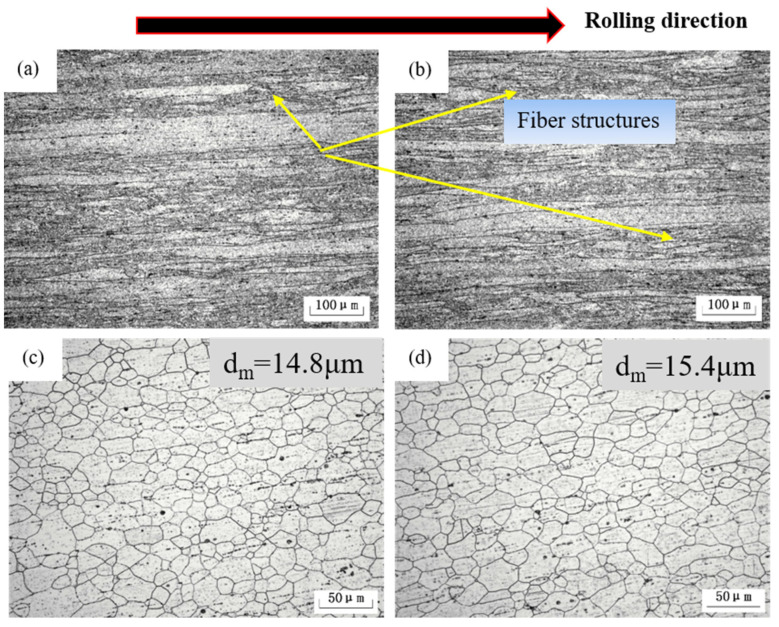
Microstructures of (**a**) as-rolled Al-4Mg alloy, (**b**) as-rolled Al-4Mg-0.6Ag alloy, (**c**) as-quenched Al-4Mg alloy and (**d**) as-quenched Al-4Mg-0.6Ag alloy.

After rolling, as shown in Figure 2a,b, a large number of fiber structures can be detected. Obviously, fiber structures are nearly parallel to the rolling direction (RD), and fine recrystallized grains nucleate within the fiber bands after solution treatment, as shown in Figure 2c,d. Most of the grains are about 10–20 μm in size and there is little difference in grain size between the two alloys. This reflects that Ag has little effect on grain refinement. The main role of Ag-addition comes from the numerous fine-scale Ag-containing phases distributed along rolling direction, which can promote work hardening. In addition, the Ag-containing particles can dissolve into the matrix easily during solution treatment and provide oversaturation conditions for subsequent aging treatment.

### 3.2. Mechanical Properties and Precipitation Process

**Figure 3:**
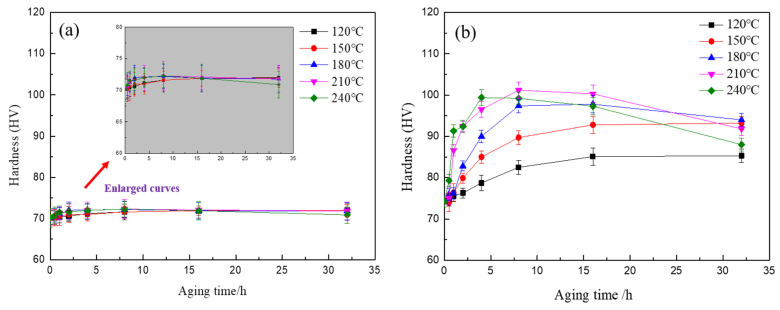
Hardness curves of (**a**) Al-4Mg alloy, (**b**) Al-4Mg-0.6Ag alloy aging at different temperatures.

Figure 3 shows the hardness curves of the two alloys aging at different temperatures. For the Ag-free alloy, the hardness changes slightly with aging temperatures and times, as shown in Figure 3a. However, as shown in Figure 3b, the age hardening response is significantly enhanced by the addition of Ag. The hardness of the Al-4Mg-0.6Ag alloy increases sharply with aging temperatures and times. This reflects that there must be some particles precipitated in the aging process, which leads to the increasing of the hardness of the alloy. Additionally, the higher the temperature is, the faster the hardness increases, the shorter the time to reach the peak hardness. In the aging process, the nucleation, growth and coarsening of precipitates are controlled by the diffusion of vacancies and solute atoms. The effect of temperature on precipitates can be expressed by the Arrhenius equation: D = D_0_exp(−Q/RT) [16]. The diffusion coefficient increases with aging temperatures. Thus, the higher the aging temperature is, the faster the atoms diffuse and the more rapidly the hardness increases. By comparing the hardness curves of the Al-4Mg-0.6Ag alloy at different aging temperatures, the best aging temperature is 210 °C.

**Figure 4:**
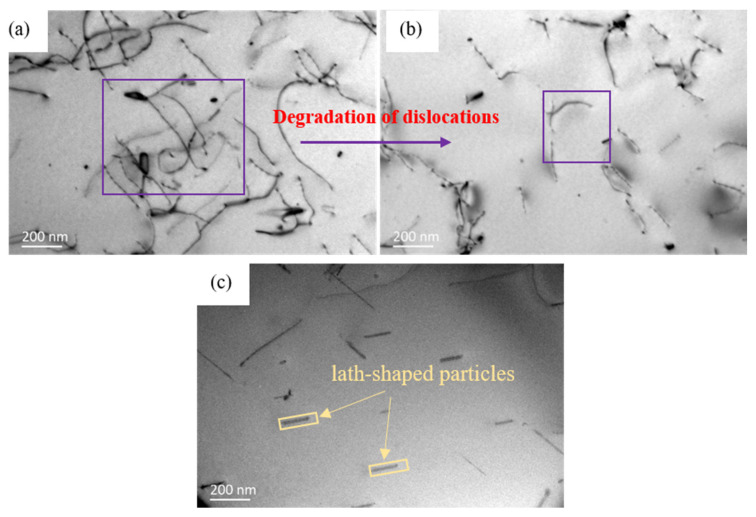
TEM micrographs of Al-4Mg alloy aged for (**a**) 1 h, (**b**) 8 h, (**c**) 32 h at 210 °C, respectively.

**Figure 5:**
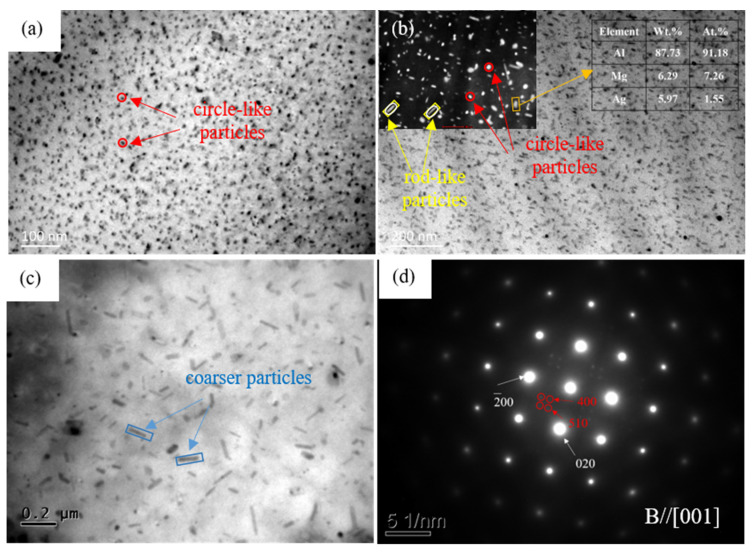
TEM micrographs of Al-4Mg-0.6Ag alloy: (**a**) aged for 1 h, (**b**) aged for 8 h, (**c**) aged for 32 h at 210 °C, respectively; (**d**) SAED pattern of the rod-like particles.

In order to illustrate the effect of Ag on the precipitation strengthening mechanism of Al-Mg alloys, TEM images of the aged Al-4Mg (-0.6Ag) alloys are shown in Figure 4 and Figure 5. For the Al-4Mg alloy, at the early aged stage (210 °C/1 h), the microstructure contains massive dislocations, forming dislocation tangles. There is no precipitated particle in the matrix, as shown in Figure 4a. When aged for 8 h at 210 °C, with the movement of vacancies and dislocations, the opposite dislocations offset each other and the amount of dislocations decrease. There is no particles at this stage, as shown in Figure 4b. At the over-aged stage (210 °C/32 h), in Figure 4c, some lath-shaped particles with dimension of 0.1–0.2μm distribute in the matrix sparsely. According to the morphology of the particles and the precipitation sequence of Al-Mg alloys [4], the lath-shaped particles can be defined as β’ with the component of Al_3_Mg_2_. For Al-Mg alloys, the clusters of Mg atoms are unstable and prefer to dissolve at artificial aging temperature. Due to the lack of nucleation sites, it is difficult for nanoparticles to precipitate uniformly. Only few coarse scale β’ phases distribute in the matrix sparsely. Due to the coarse scale and discrete distribution, the β’ phase has little effect on the strengthening of Al-Mg alloys.

In the aging process, the size, distribution and type of the precipitates change significantly, compared with the Ag-free alloy. At the early stages (210 °C/1 h), in Figure 5a, numerous nanoscale circle-like particles distribute in the matrix uniformly and they can be identified as GP zones according to their dimension and aging condition. The GP zones can be speculated as the accumulation of Mg and Ag atoms, as the two kinds of atoms had strong binding energy [17,18]. At the peak-aged stage, the circle-like GP zones transformed into the MgAg phase, which may have independent lattice structure. The circle-like MgAg phase promotes the aging strengthening effect of Al-Mg-Ag alloy efficaciously. Some circle-like particles transformed into rod-like phases and the microstructures contained both circle-like and rod-like particles, which formed multiscale coexistence phase structures. This multi-size structures can hinder and twine the dislocations efficaciously in the plastic deformation process and improve the strength of the alloy. The corresponding selected area electron diffraction (SAED) pattern in Figure 5d indicates that the rod-like particles have a body-centered cubic structure with a lattice parameter of a = 1.41 nm. A higher magnification image of the precipitates is shown in the top left corner of Figure 5b. The EDS results of the rod-like particles indicate that this phase contains elements of Al, Mg and Ag. Based on the results above, the rod-like particles can be speculated as T phase with a composition of (Mg_32_(Ag, Al)_49_. The results are also in agreement with the high-Mg content of the Al-Mg-Ag alloys [9,19]. At the over-aged stages, with the increasing aging times, the size of the precipitates keeps on coarsening and the amount decreases, as shown in Figure 5c. The effect of precipitates on hindering the movement of dislocations is weakened. As a result, the hardness of the alloy decreases.

**Figure 6:**
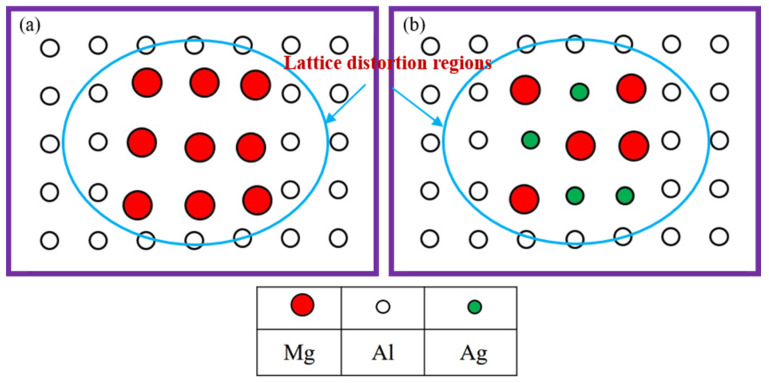
Diagram of the atomic aggregation of the alloys without (**a**) and with (**b**) Ag addition.

For Al-Mg alloys, the formation of GP zones is attributed to the movement of vacancies. Therefore, the gathered solute atoms must be coherent with the matrix. If the atomic volume of precipitates and matrix varied widely (Al is 2.862 Å and Mg is 3.196 Å), as shown in Figure 6a, the matrix and precipitates will produce lattice distortion. Therefore, the influence of Mg atoms on the lattice constant of the Al matrix is conspicuous. The elastic strain energy between GP zones and aluminum matrix is large and Gibbs-free energy improves. GP zones are unstable and transformed to equilibrium phase quickly. Therefore, it is difficult to detect GP zones in the artificial aging process of Al-Mg alloys. Due to the absence of GP zones, which act as nucleation sites for the transient phases, the aging strengthening effect is limited [20,21].

For the Ag-addition alloy, Ag modifies the precipitation process from the earliest stage of the decomposition through a preferred Mg-Ag interaction. After the addition of Ag, the precipitation sequence of Al-4Mg-0.6Ag alloy in this passage can be summarized as follows: GP zones → MgAg phase → T phase (Mg_32_(Ag, Al)_49_). After the addition of 0.6 wt.% Ag in the Al-4Mg alloy, as the atomic radii of Al and Ag differ by only 0.5% [22], the lattice distortion of GP zones is alleviated and the elastic strain energy between GP zones and aluminum matrix reduces, as shown in Figure 6b. Therefore, the GP zones can exist in the matrix stably. In addition, Ag is easier to bond with Mg and increased the amount of vacancy, which promotes the formation of GP zones [23]. Numerous GP zones provide nucleation sites for the nanoscale dispersed precipitates. As a result, the strength of the Al-4Mg alloy is improved by the addition of Ag.

**Figure 7:**
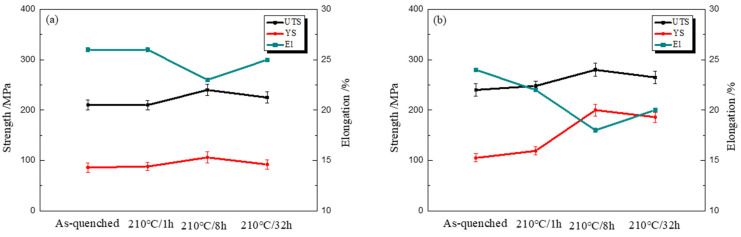
Mechanical properties of (**a**) Al-4Mg alloy, (**b**) Al-4Mg-0.6Ag aged for different times at 210 °C.

**Figure 8:**
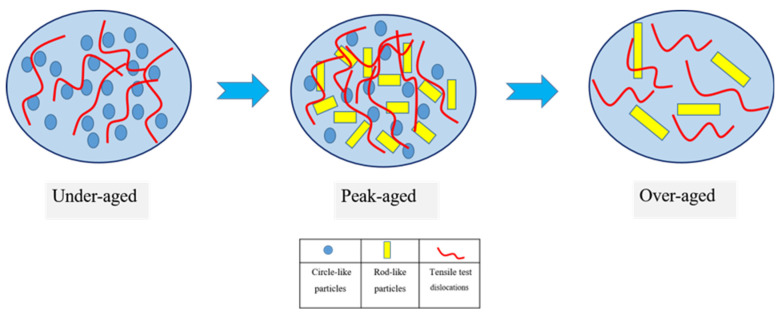
Schematic illustration showing the effects of precipitates on hindering dislocations of the Ag-bearing alloy.

Figure 7 presents the mechanical properties of the two alloys under each aging condition. As the uniformly distributed precipitates can impede dislocation movement in plastic deformation process, the strength of the aged Al-4Mg-0.6Ag alloy is visibly improved. The effects of the nanoscale precipitates on hindering the movement of dislocations are shown in Figure 8. At the under-aged stage of the Ag-bearing alloy, the dislocations can pass the coherent precipitates by cutting or bypassing in the plastic deformation process. At the peak-aged stages (210 °C/8 h), the circle-like and rod-shaped particles with different dimensions and orientations can heavily impede the dislocations, forming dislocation stacking. However, for the Ag-free alloy, the dislocation slip is easier, as no precipitates are detected. Therefore, at the peak-aged stage, the ultimate tensile strength (UTS) and yield strength (YS) of the Ag-bearing alloy increased by 40 MPa and 94 MPa, which increased by 16.7% and 88.7% than that of the Ag-free alloy, respectively. At the over-aged stage of the Ag-containing alloy, precipitates become coarser and the amount of which decreases. The hindrance of precipitates on dislocations is weakened, which leads to the reduction in the strength of the alloy.

**Figure 9:**
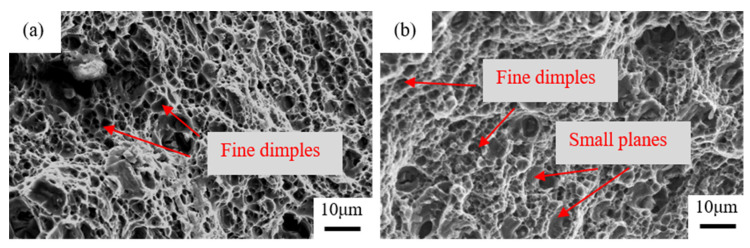
Fracture surfaces of the two alloys aged for 8 h at 210 °C: (**a**) Al-4Mg alloy, (**b**) Al-4Mg-0.6Ag alloy.

Figure 9 shows the tensile fracture surfaces of the peak-aged samples of the two alloys. The fracture surfaces of each sample show ductile rupture with characteristic dimples. In Figure 9a, the dimples of the Ag-free alloy are tiny and uniform, which manifests excellent ductility of the alloy. However, for the Ag-bearing alloy, in addition to the fine dimples, some small planes can also be detected, as shown in Figure 9b, which leads to the ductility degradation. This can be explained as follows: for the Al-4Mg alloy, there are fewer barriers to dislocation slip and the dislocations can overcome the petty deformation resistance and pass through crystal readily in the process of plastic deformation. For the Ag-bearing alloy, the dislocations cannot pass through the particles and lead to dislocations stacks. The dislocations stacks bring about stress concentration and reduce the dislocation storage capacity. As a result, the work-hardening rate decreases and the ductility is deteriorated.

## 4. Conclusions

In this paper, the influence of Ag on microstructure, precipitation behavior and mechanical properties of Al-4Mg alloy are investigated systematically. Detailed results are as follows:

(1) For the as-cast Al-4Mg alloy, the addition of Ag can reduce the dimension of the precipitates and promote the precipitation of numerous fine-scale AlMgAg particles.

(2) During aging, Ag promotes the precipitation of the nanoscale (Mg_32_(Ag, Al)_49_. The best aging temperature was 210 °C.

(3) For the peak-aged stage, the UTS of the Ag-bearing alloy was 280 MPa and the YS is 200 MPa, which is 40 MPa and 94 MPa higher than that of the Ag-free alloy, respectively.

## Figures and Tables

**Table 1:** Composition of test points as marked in Figure 1 (wt.%).

Position	Mg	Ag	Fe	Si	Al
001	0.95		31.96		67.09
002	4.39		15.98		79.63
003	4.16		21.05		74.79
004	1.12	0.82	32.72		65.33
005	2.26	53.51	3.23	1.83	39.17
006	6.97	23.31		0.91	82.65
007	17.64	28.92		1.37	52.07

## Data Availability

The raw/processed data required to reproduce these findings cannot be shared at this time as the data also forms part of an ongoing study.
